# What is the appropriate size criterion for proton radiotherapy for hepatocellular carcinoma? A dosimetric comparison of spot-scanning proton therapy versus intensity-modulated radiation therapy

**DOI:** 10.1186/1748-717X-8-48

**Published:** 2013-03-05

**Authors:** Chie Toramatsu, Norio Katoh, Shinichi Shimizu, Hideaki Nihongi, Taeko Matsuura, Seishin Takao, Naoki Miyamoto, Ryusuke Suzuki, Kenneth Sutherland, Rumiko Kinoshita, Rikiya Onimaru, Masayori Ishikawa, Kikuo Umegaki, Hiroki Shirato

**Affiliations:** 1 Department of Medical Physics, Hokkaido University Graduate School of Medicine, Sapporo Japan; 2 Department of Radiation Medicine, Hokkaido University Graduate School of Medicine, Kita-15Nhisi-7, , Kita-ku, Sapporo 060-8638, Japan

**Keywords:** Spot-scanning proton therapy, Intensity-modulated radiation therapy, Hepatocellular carcinoma, Radiation induced liver disease

## Abstract

**Background:**

We performed a dosimetric comparison of spot-scanning proton therapy (SSPT) and intensity-modulated radiation therapy (IMRT) for hepatocellular carcinoma (HCC) to investigate the impact of tumor size on the risk of radiation induced liver disease (RILD).

**Methods:**

A number of alternative plans were generated for 10 patients with HCC. The gross tumor volumes (GTV) varied from 20.1 to 2194.5 cm^3^. Assuming all GTVs were spherical, the nominal diameter was calculated and ranged from 3.4 to 16.1 cm. The prescription dose was 60 Gy for IMRT or 60 cobalt Gy-equivalents for SSPT with 95% planning target volume (PTV) coverage. Using IMRT and SSPT techniques, extensive comparative planning was conducted. All plans were evaluated by the risk of RILD estimated using the Lyman-normal-tissue complication probability model.

**Results:**

For IMRT the risk of RILD increased drastically between 6.3–7.8 cm nominal diameter of GTV. When the nominal diameter of GTV was more than 6.3 cm, the average risk of RILD was 94.5% for IMRT and 6.2% for SSPT.

**Conclusions:**

Regarding the risk of RILD, HCC can be more safely treated with SSPT, especially if its nominal diameter is more than 6.3 cm.

## Background

Unresectable primary and metastatic liver cancer is a frequent cause of morbidity and mortality. Focal liver radiotherapy (RT) can be used as a treatment option and technological advancements have facilitated the safe use of highly dose-conformal RT in liver cancers. However, RT for large liver cancers is still challenging because of the liver’s low tolerance dose for radiation-induced liver disease (RILD) [1,2]. RILD is the most common liver toxicity following RT [3,4]. Sparing of normal tissue (normal liver) is severely required, and this limits the role of RT for treatment of unresectable hepatic malignancies. The widespread availability of intensity-modulated radiation therapy (IMRT) allows one to achieve significant improvements in dose distributions for partial liver irradiation. IMRT inverse planning can generate complex spatial dose distributions that closely conform to the target while sparing the organ at risk (OAR). However, the downside to IMRT is the larger volume of normal tissue exposed to lower radiation doses. This can increase the odds of developing RILD [5].

Proton beams are known to have superior normal tissue sparing compared with photons. Protons have a finite range, which generally leads to an improved dose distribution, and this physical property can be used more effectively for the treatment of large volume tumors when they are surrounded by critical organs [6]. Sugahara et al. reported that proton RT was effective and safe for patients with hepatocellular carcinoma (HCC) greater than 10 cm in maximal dimensions [7].

Although proton RT has an advantage compared with photon RT, to the best of our knowledge, there are no available data on the appropriate size criterion for radiotherapy using proton beams for HCC. In this study, we performed a dosimetric comparison of IMRT and spot-scanning proton therapy (SSPT) for HCC to investigate the impact of tumor size on the risk of RILD using the normal-tissue complication probability (NTCP) model.

## Methods and materials

### Patients

We obtained approval from our institutional review board at Hokkaido University Hospital for this retrospective dosimetric study. Between January and October 2011, 10 consecutive patients with HCC treated at our institute, with GTV greater than 6 cm in diameter and portal vein tumor thrombosis (PVTT), were included in this study. The tumor size and that location data are summarized in Table 1. CT data sets were acquired using a slice thickness of 2 or 2.5 mm. All the patients received X-ray radiotherapy targeting the PVTT alone, not the whole tumor, as a palliative treatment.

**Table 1:** Targets and normal liver dimensions

**Plan ID.**	**Main location of tumor**	**PTV (cm**^**3**^**)**	**GTV (cm**^**3**^**)**	**Max. diameter of GTV (cm)**	**Nominal diameter of GTV (cm)**	**Normal liver (cm**^**3**^**)**	**Overlapping of PTV and normal liver (cm**^**3**^**)**
1	The right main branch of the portal vein	204.7	20.1	4.7	3.4	1660.6	142.0
2	The main trunk and the main branches of the portal vein	348.0	59.2	7.2	4.8	2645.6	189.0
3	The main trunk and the main branches of the portal vein	341.6	70.3	8.5	5.1	2658.7	302.3
4	Segment 3, 4	381.7	82.1	6.8	5.4	1783.5	207.8
5	Segment 4, 8	405.9	130.9	7.3	6.3	814.6	300.3
6	Segment 1, 4, 6, 7, 8	844.8	245.8	15.1	7.8	1312.6	310.2
7	Segment 7, 8	764.2	284.2	12.4	8.2	1216.5	367.3
8	Segment 3, 4, 5, 7, 8	785.6	299.3	10.3	8.3	1339.9	411.2
9	Segment 3, 4, 5, 7, 8	848.9	344.5	13.8	8.7	1676.9	399.7
10	Segment 6, 7, 8	1488.3	720.6	15.2	11.1	965.4	489.4
11	Segment 1, 5, 6, 7, 8	1822.4	916.0	15.4	12.1	1088.7	563.2
12	Segment 1, 4, 5, 6, 7, 8	2222.6	1638.9	18.0	14.6	1087.5	621.7
13	Segment 1, 4, 5, 6, 7, 8	3094.2	2194.5	22.0	16.1	629.2	630.1
Mean	-	1042.5	539.0	-	8.6	1452.3	379.6

### Treatment planning

In order to compare large volume irradiation, a GTV encompassing the whole tumor was re-contoured by a radiation oncologist using one of two treatment planning systems: (TPS) XiO (CMS Inc., St Louis, MO), Pinnacle^3^ (Philips, Inc., Madison, WI) or Eclipse TPS Ver.10.0.0 (Varian Medical Systems, Palo Alto, CA). The clinical target volume (CTV) was defined as a 5 mm expansion of the GTV minus areas of overlap with uninvolved extra hepatic structures (e.g., lung, abdominal wall, intestine) [8]. A 1 cm margin for set-up margin and organ motion was uniformly added to the CTV to generate the planning target volume (PTV), assuming the use of gated irradiation. Target and normal structures were formatted and transferred to the Eclipse TPS Ver.10.0.0. Here, for patients with extremely large targets (PTV >1500 cm^3^), we prepared two different plans: one with the GTV contoured PVTT alone (plan ID 1, 2 and 3 in Table 1) and the other with the GTV contoured whole tumor area (plan ID 11, 12 and 13 in Table 1). This is because we were concerned that patients with large tumors should receive RT targeting the PVTT alone, even though SSPT can spare the surrounding normal liver. A total of 13 GTVs in 10 patients were used for IMRT plans and SSPT plans (total of 26 plans were generated), in this comparative study. We made a comparative dosimetric study between simulated plans of photon and proton beams on various irradiation volumes of HCC utilizing IMRT and SSPT plan techniques. These simulated plans were generated in Eclipse TPS, assuming photon treatment with an MHCL-15SP v80m (Mitsubishi Electronics Co., Ltd., Tokyo) LINAC and proton treatment with a Probeat III (Hitachi Co Ltd, Japan) proton accelerator [9], respectively.

For the IMRT plans, five to nine evenly spaced intensity modulated fields were generated with a 6 MV photon beam. For Proton plans, the SSPT technique [10], which can be generated using the inverse planning approach like IMRT, was applied. For SSPT plans, simple arrangements of two or three proton fields were selected. The dose distributions of proton plans generated with only two of three fields were dependent on field incidence, achieving good results for the normal liver spared by beam entrance. First, one beam angle was selected so that beam paths can take the shortest way to cover the target. Then one or two more beams were added and each beam’s angle was adjusted so that dose distribution was homogenous. The SSPT plans were simulated as individually weighted proton Bragg peaks distributed throughout the PTV. Energies were selected from those actually deliverable. The energies required to cover the target homogeneously were 70 to 180 MeV. For SSPT plans, a relative biologic effectiveness factor for protons of 1.1 was employed.

The dose prescription for this study was 60 Gy for IMRT plans or 60 cobalt Gy equivalents (CGE) for SSPT plans covering 95% of the PTV, delivered in 15 fractions [11,12]. The dose volume constraints for the liver used in the planning process were taken on the basis of normal tissue tolerances as estimated by Emami *et al*. [13]. The maximum doses delivered to one third and two thirds of the normal liver were planned in order not to exceed the tolerance dose (TD) 5/5 (the probability of 5% complications within 5 years from irradiation). The constraint for the normal liver was set at its volume receiving 33 Gy or CGE or more (V33) less than 67% and V42 less than 33%. We also considered the dose-volume limits for therapeutic partial liver RT which is recommended by Pan CC *et al*. [14]. Mean normal liver doses were planned in order not to exceed the tolerance dose 28 Gy (CGE) in 2 Gy or CGE fractions for primary liver cancer. Dose limits of 33, 42 and 28 Gy (CGE) in 15 fractions were specified. These are equivalent to 35, 50 and 28.6 Gy (CGE) using 2 Gy (CGE) per fraction assuming an α/β ratio of 2.5, respectively. Both IMRT and SSPT plans were optimized with the requirement that at least 95% of the PTV received the prescribed dose.

### Evaluation

Once an acceptable treatment plan was obtained, dose-volume histogram (DVH) analysis was performed. First, DVH comparisons of the IMRT and SSPT plans were made. Although many representations of the data are valid, we used the Lyman-Kutcher-Burman (LKB) NTCP model [15-17] to calculate the risk of RILD. The LKB-NTCP model is used to estimate the volume dependence of normal tissue toxicity that permits comparisons between plans based on DVHs. The Lyman-NTCP model describes the probability of a complication after uniform radiation of a fractional volume of normal tissue (*v*) to a dose (*D*), as

(1) $$\text{NTCP}=\phi \left(t\right)=\frac{1}{\sqrt{2\pi }}\int_{-\infty }^{t}\text{exp}\left(-,\frac{x^{2}}{2}\right)\mathrm{dx}$$

where

(2) $$t=\left(D,-,\frac{TD_{50}\left(v\right)}{m\cdot TD_{50}\left(v\right)}\right)$$

*TD*_*50*_(*v*) represents the tolerable dose associated with a 50% chance of complications for uniform partial liver irradiation, where *TD*_*50*_(*v*) is related to the whole liver (*v* = 1) tolerance through the power law relationship:

(3) $$TD_{50}\left(v\right)=TD_{50}\left(1\right)\cdot v^{-n}$$

*TD*_*50*_(1) represents the tolerance of the whole organ to irradiation, *m* characterizes the steepness of the dose-response at *TD*_*50*_(1), and *n* represents the volume effect, which relates the tolerance doses of uniform whole organ irradiation to uniform partial organ irradiation. Dawson and colleagues derived these three parameters as n = 0.97, m = 0.12, and *TD*_*50*_(1) = 39.8 Gy (CGE) (hereafter referred to as the Michigan parameters) from the LKB-NTCP model fitted to the complication data for 203 patients with liver cancer treatment, 11 fr/wk, 1.5–1.65 Gy (CGE) /fraction [3]. The median dose of radiation delivered was 52.5 Gy (CGE) (range 24–90). In this study, the risk of RILD was estimated using the LKB-NTCP model with the Michigan parameters.

### DVH normalization

To correct for the difference in protocol between this study and the Michigan study [3], normal liver DVHs were normalized before computation of the dose distributions from which the DVHs were computed. The physical dose values of each plan were converted to normalized iso-biologic effective doses using the linear quadratic model (LQ-model). That is, the normal liver cumulative DVH dose bins were converted to a fraction-size equivalent dose (*FED*) [18] described as

(4) FEDαβfs≡nd1+dαβ1+fsαβ

where *f*_*s*_ denotes a reference fraction size. For example, the dose per fraction delivered using the schedule from which the modeled NTCP data was derived, *d*, is the physical dose per fraction, *n* is the total number of fractions, and α/β is the ratio of the linear LQ-model parameters for the organ and end point at risk. The mean *FED* is then described as

(5) MeanFEDαβfs=∑n=1NbFEDαβfsivi

where *N*_*b*_ denotes the total number of dose bins in the differential DVH, *FED*^*fs*^_α/β__*i*_ is the *FED* in the *i*-th dose bin, and *v*_*i*_ is the partial volume associated with the *i*-th dose bin (*∑*^*Nb*^_*n=1*_*v*_*i*_ =1). In this study, we calculated the *FED*, continuing to work with the reference point dose per fraction *f*_*s*_ and α/β ratio for which Dawson’s NTCP model fit derived, following the treatment schedule employed by Dawson *et al*. [3], and using an α/β = 2.5.

### Effective volumes

To convert the non-uniform complex dose distributions into “equivalent” uniform dose distributions, the Kutcher-Burman effective volume (V_eff_)-DVH reduction scheme [16] was used. The V_eff_ method transforms the histogram into a uniform histogram of height V_eff_ and dose, D_max_, equal to the maximum dose in the histogram. This transformed histogram is assumed to indicate the same probability of complications as the original histogram. Each step in the histogram of height Δ*v*_i_ and extension D_i_ is assumed to satisfy a power law relationship so that it is adjusted to one of smaller volume ΔV_eff_ and extension D_max_ using

(6) ΔVeffi=ΔviDiDmax1n

where n is a size parameter. This procedure is applied to each bin of the histogram. The normalized normal liver DVH with a reduction of the DVH to the effective liver volume is then irradiated as

(7) Veff=Δvmax+Δv1D1 Dmax1n+Δv2D2Dmax1n+⋯

The LKB-NTCP was then calculated using the Michigan parameters and the normalized normal liver DVH with a reduction of the DVH to the V_eff_.

### Statistical analysis

Mean *FED*, V_eff_ and the risk of RILD for normal liver were calculated and compared between IMRT plan and SSPT plan. We investigated the size of GTV that could be delivered at a prescribed dose for targets with successful achievement of dose constraints to the normal liver. Assuming all GTVs were spherical, the nominal diameter was calculated and used in this study. The median volume of GTV was 539.0 cm^3^ (range, 20.1–2194.5) and the median nominal diameter was 8.6 cm (range, 3.4–16.1) as shown in Table 1.

The mean *FED* for normal liver was checked against the Michigan study [3] in which Dawson *et al*. [3] reported that no cases of RILD were observed when the mean liver dose was under 31 Gy, and the mean liver doses associated with a 5% risk of RILD for patients with metastatic and primary liver cancer are 37 Gy and 32 Gy, respectively (in 1.5 Gy per fraction, assuming an α/β = 2.5 for the liver). The values of mean *FED*, V_eff_ and the risk of RILD for normal liver were aligned as a function of the nominal diameter of GTV. For the values of mean *FED* and V_eff_, the Wilcoxon signed-rank test was performed to compare differences between IMRT and SSPT plans, with a *p* value of < 0.05 being considered significant.

## Results

### DVH comparison among plans

**Figure 1:**
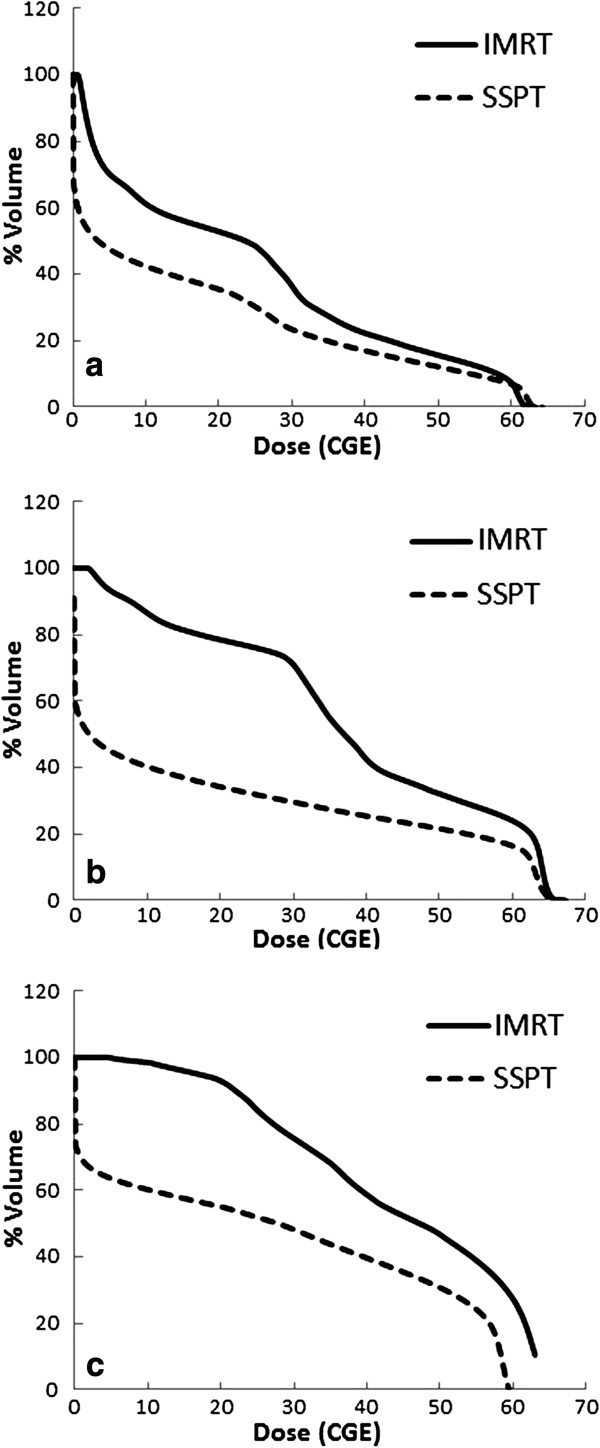
**Cumulative dose-volume histograms (cDVH) of normal liver for IMRT plans (solid line) and SSPT plans (dashed line). **(**a**) cDVH for patients with nominal diameter of GTV (**a**) 5.1 cm, (**b**) 7.8 cm and (**c**) 16.1 cm. Triangles in each figure were dose constrains for one-third and for two-thirds of the volume of the normal liver.

**Figure 2:**
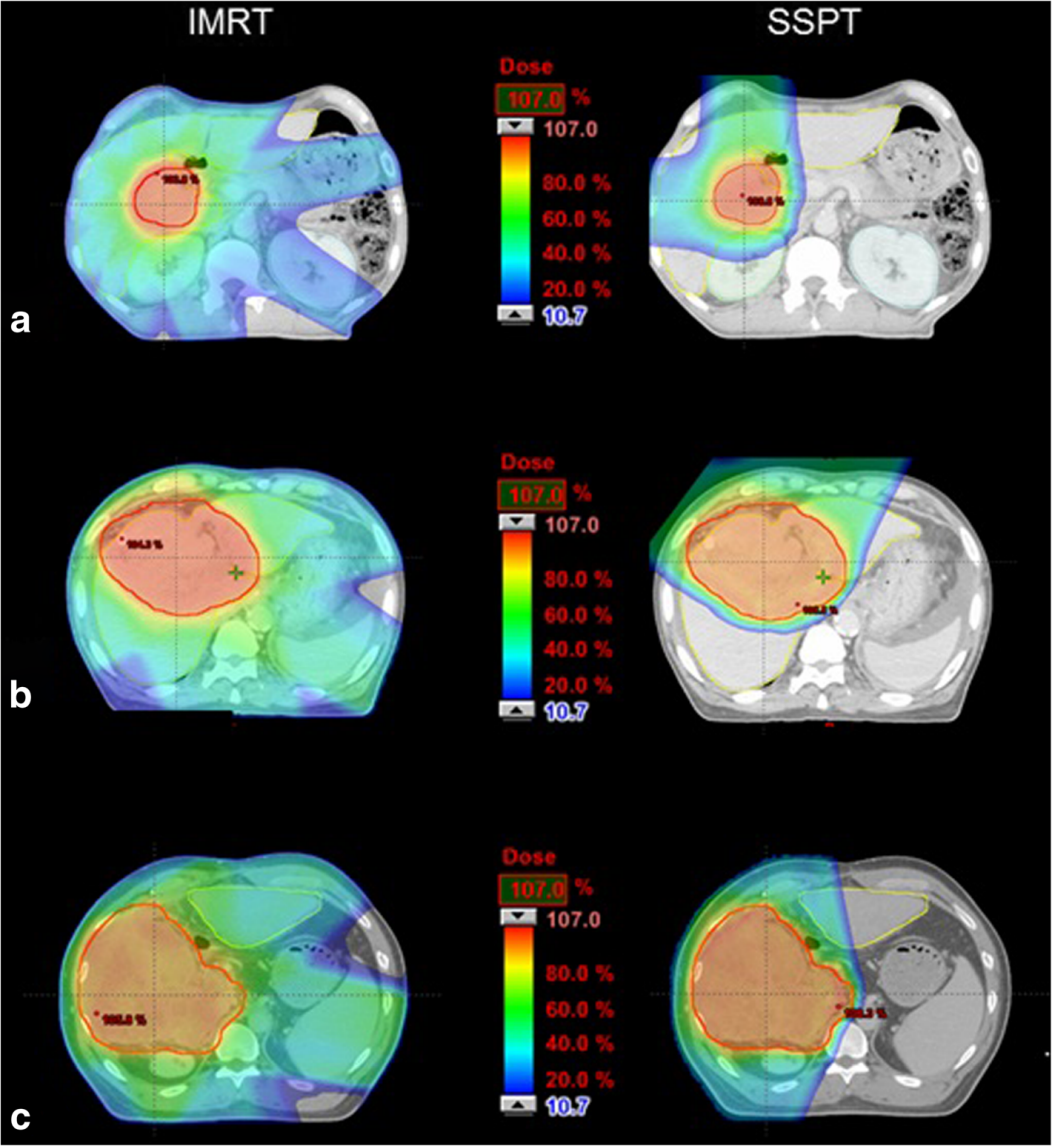
Dose distributions obtained with IMRT plans and SSPT plans for patients with nominal diameter GTV of (a) 5.1 cm, (b) 7.8 cm and (c) 16.1 cm.

For all 10 patients, a total of 26 plans for each technique of IMRT and SSPT were evaluated through careful review of the PTV isodose distributions on all slices by a radiation oncologist. Each plan provided good coverage of the target, covering 95% of the PTV. For the patients with relatively small targets having nominal diameters less than or equal to 6.3 cm (volume of GTV ≤ 130.9 cm^3^), the target dose optimization according to dose constraints to one third and two thirds of the volume of the normal liver was successfully achieved for IMRT plans (Table 2). However, when the nominal diameter was greater than or equal to 7.8 cm, none of the IMRT plans were able to deliver a prescribed dose of 60 Gy covering 95% of the PTV for larger targets without sacrificing normal liver. SSPT plans achieved dose constraint for the normal liver successfully in all plans except for the plan with a 16.1 cm nominal diameter GTV (Table 2). SSPT plans obtained clearly superior results to IMRT plans for every case. Examples of the DVH and dose distributions obtained with IMRT and SSPT plans for the cases of nominal diameter of GTV = 5.1 cm (a), 7.8 cm (b) and 16.1 cm (c) are shown in Figure 1 and Figure 2.

**Table 2:** **Values of V**_**eff **_**and mean *FED***

**ID.**	**Nominal diameter of GTV (cm)**	**V33**		**V42**		**V**_eff_^*^			**D**_**Mean**_		Mean FED^*^		
		**IMRT (%)**	**SSPT (%)**	**IMRT (%)**	**SSPT (%)**	**IMRT**	**SSPT**	**p-value**	**IMRT (Gy)**	**SSPT (Gy)**	**IMRT (Gy)**	**SSPT (Gy)**	**p-value**
1	3.4	21.4	19.3	18.1	15.9	0.56	0.29	0.021	24.4	16.3	35.9	20.2	0.012
2	4.8	26.4	19.1	20.8	14.2	0.49	0.30		21.4	16.4	33.1	20.1	
3	5.1	18.7	15.9	15.5	14.5	0.46	0.25		19.8	13.6	30.3	16.6	
4	5.4	25.4	17.8	17.9	17.9	0.40	0.24		24.2	17.7	32.8	15.2	
5	6.3	31.2	21.2	23.2	20.2	0.47	0.33		37.9	22.4	37.9	19.9	
6	7.8	61.8	30.0	40.9	28.3	0.70	0.47	< 0.001	36.9	27.4	52.5	33.1	< 0.001
7	8.2	67.5	27.3	59.7	27.3	0.72	0.49		36.1	28.1	54.5	32.5	
8	8.3	76.9	41.2	42.7	27.7	0.62	0.32		32.7	17.4	56.7	24.2	
9	8.7	70.0	45.6	60.4	27.9	0.75	0.53		38.3	28.6	58.1	33.3	
10	11.1	61.3	39.8	60.2	28.2	0.76	0.44		38.8	24.4	67.9	27.3	
11	12.1	89.5	50.9	67.3	30.9	0.83	0.57		41.9	31.7	75.4	32.4	
12	14.6	70.9	48.2	55.3	29.7	0.73	0.45		37.1	25.6	64.2	30.4	
13	16.1	64.7	48.5	62.4	37.9	0.86	0.55		42.4	30.3	78.4	31.5	
ave.	8.6	52.7	32.7	41.9	24.7	0.64	0.40		33.2	23.4	52.1	25.9	

*The values of V_eff _and mean *FED *were normalized to 1.5 Gy per fraction, assuming an α/β of 2.5.

### Estimation of RILD

The mean *FED* of normal liver, summarized in Table 2, increased with the size of the GTV. The averages of the mean *FED* of normal liver for IMRT plans and SSPT plans were 52.1 Gy (range, 30.3–78.4) and 25.9 CGE (range, 15.2–33.3), respectively. SSPT plans maintained a lower mean *FED* of normal liver that of IMRT plans (*p* =*0*.*01*) The mean *FED*s for SSPT plans reached a plateau at around 30 CGE, while that for IMRT plans increased with the size of the tumor. The values of V_eff_ for each plan are also summarized in Table 2. The mean values of V_eff_ for normal liver in IMRT plans and SSPT plans were 0.64 (range, 0.40–0.86) and 0.42 (range, 0.29–0.60), respectively. SSPT achieved a lower V_eff_ for normal liver than did IMRT ( *p* < *0*.*001* ). The V_eff_ for normal liver of the SSPT reached a plateau around 0.5 even if the size of the tumor increased while that for IMRT plans increased with tumor size. For IMRT plans, both the mean *FED* and V_eff_ for normal liver drastically increased from between 6.3–7.8 cm nominal diameter of GTV, and the difference between IMRT and SSPT plans was significant when the nominal diameter of GTV was more than 6.3 cm (Table 2).

**Figure 3:**
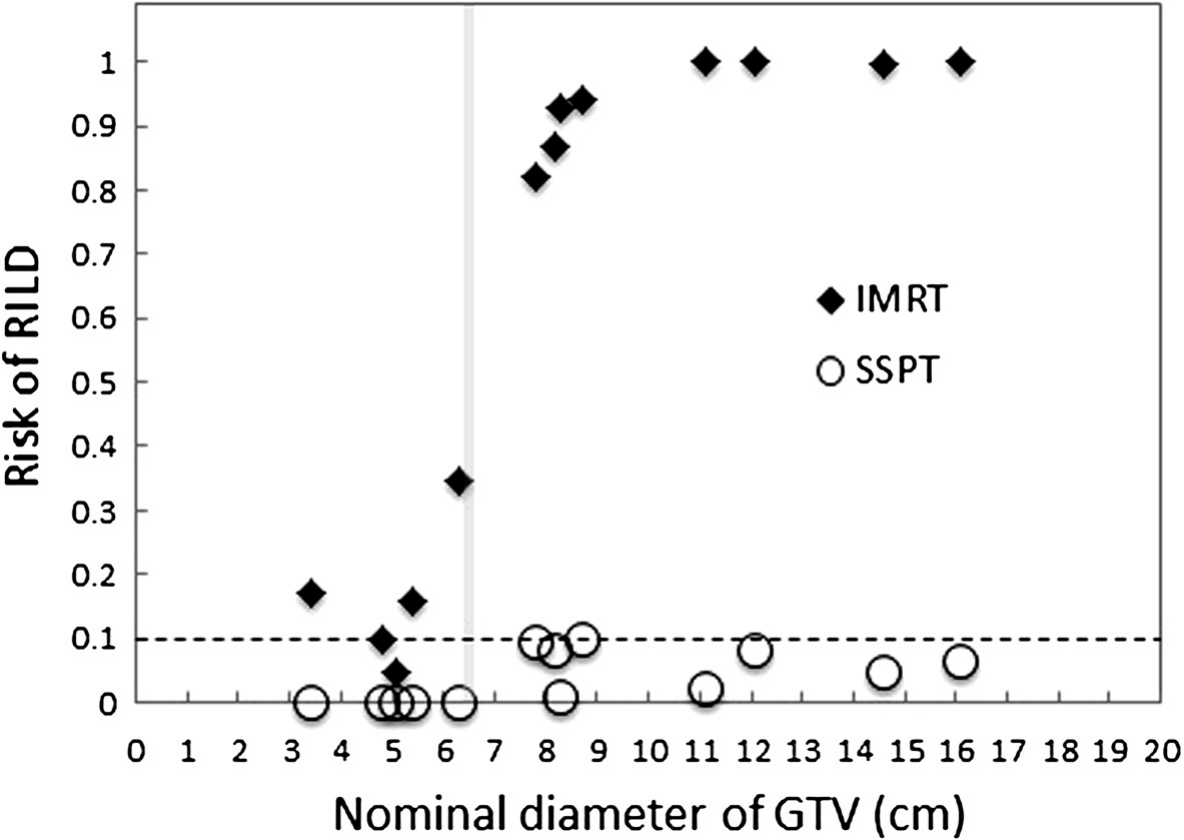
**Relationship of the risk of RILD to the nominal diameter of GTV. **Prescribed doses of 60 Gy for IMRT (black diamond) and 60GyE for SSPT (open circle), delivered in 15 fractions, were normalized for all plans to 1.5 Gy b.i.d. assuming an α/β of 2.5, based on the LKB-NTCP model (n = 0.97, m = 0.12, *TD*_*50*_(1) = 39.8 Gy ). The difference of the risk of RILD between IMRT and SSPT plans clearly increased from 6–7 cm nominal diameter of GTV.

Figure 3 shows the relationship between risk of RILD probability and tumor size. As expected, the risk of RILD probability for HCC for all plans increased in relation with the value of V_eff_ and mean *FED* for normal liver; that for IMRT varied from 0.03 to 1.00, while that for SSPT varied from 0.0003 to 0.087. IMRT plans were able to keep the risk of RILD low in cases of GTV nominal diameter below 6.3 cm, but then the risk increased drastically with tumor size. For tumors of GTV nominal diameter less than or equal to 6.3 cm, the averages of RILD probability for IMRT and SSPT plans were 0.016 and 0.009, respectively. For GTV nominal diameter greater than 6.3 cm, the average RILD probabilities for IMRT and SSPT plans were 0.942 (range, 0.822–1.00) and 0.045% (range, 0.001–0.087), respectively. The RILD probabilities were lower than IMRT when using SSPT for all cases, and they were significantly lower for nominal diameters of GTV greater than 6.3 cm.

## Discussion

In this study, the SSPT technique was applied. The scanning target volume, an optimization volume for SSPT planning, should be defined for each liver cancer patient using the distal margin including the range uncertainties defined by *d*_*m*_ = 0.035 × *R* + *U*, where *R* is the most distal range in cm for the CTV, and *U* is the beam range uncertainty [19-21]. The beam range uncertainty for accelerator energy and for pre-absorber devices [22] should be considered here, and expansions in any direction should be defined based on our own experience of setup uncertainties, which would be similar to the margins used for IMRT [23]. Because our proton therapy system is under construction these investigations of range uncertainty and margin for this area will be presented in a future publication. In this study, a 1 cm margin for set-up margin and organ motion was uniformly added to CTV to generate PTV. This margin recipe was based on a previous article [24].

Even using SSPT, the risk of RILD was sometimes above 5% in this study. With the application of a real-time tumor tracking (RTRT) system [25] this margin can be decreased. The combined SSPT and RTRT system is expected to permit safe treatment of large moving liver cancers. Investigation of the proper margin to generate PTV from CTV in cases of SSPT with the application of RTRT also will be a topic of future study.

In this study, estimations of RILD were calculated using the LKB-NTCP model with the Michigan parameters. However, inherent biologic uncertainties are present in all NTCP models. As the prescription for proton therapy is very different from that used with the liver for which the NTCP model was developed, the protocol used in this study is different from the Michigan parameters [3]. This study could not confirm the accuracy of the risk of RILD, and we should note the possibility that patients may have an impaired liver function because of an underlying cirrhosis in the course of HCC and therefore even sub-RILD doses may lead to a severe deterioration of the liver function [26,27]. Future work is required to better understand the partial volume tolerance of the liver to SSPT. However, the risk of RILD for the SSPT plan is confirmed to be low enough, and the estimations in this study are still useful for safely guiding irradiation volume allocation for clinical treatment cases. This study should help to expand the understanding of dose response once mature outcome data are available.

The risk of RILD may not only depend on tumor size but also on the location, due to the integral dose to normal liver tissue in the entrance channels for proton fields. In the clinical case, it is also required to evaluate the safety of beam arrangement, prescription and fractionation schemes considering the tumor location. In this study, we used unbiased data on tumor localization as summarized in Table 1. Beam angles were selected simply in terms of sparing healthy liver tissue. In this dosimetric study, we investigated the impact of tumor size itself on the risk of RILD. Our results show that if the nominal diameter of GTV is more than 6.3 cm, average of the risk of RILD was 94.5% while that of SSPT was 6.2%. Although the advantage of protons in sparing the normal liver has been reported in several papers [6,7], as far as we could survey, this is the first report to investigate the impact of tumor size on the risk of RILD, offering a clear reason why the advantage of proton therapy becomes effective from a nominal diameter GTV of around 6 cm.

## Conclusions

A comparative dosimetric study was undertaken between SSPT and IMRT. All plans were evaluated with DVH-analysis and the risk of RILD was estimated. Regarding the risk of RILD, HCC can be more safely treated with SSPT, especially if its nominal diameter is more than 6.3 cm.

## Abbreviations

SSPT: Spot-scanning proton therapy; IMRT: Intensity-modulated radiation therapy; HCC: Hepatocellular carcinoma; RILD: Radiation induced liver disease; GTV: Gross tumor volumes; CTV: Clinical target volume; PTV: Planning target volume; NTCP: Normal-tissue complication probability; PVTT: Portal vein tumor thrombosis; TPS: Treatment planning systems; CGE: Cobalt Gy equivalents; TD: Tolerance dose; DVH: Dose-volume histogram; LKB-NTCP: Lyman-Kutcher-Burman the normal-tissue complication probability.

## Competing interests

There are no actual or potential conflicts of interest to disclose for any of the authors.

## Authors’ contributions

CT reviewed and analyzed the data, performed statistical analyses, created the figures, and drafted the manuscript. NK reviewed and analyzed the data, performed statistical analyses, and assisted in drafting the manuscript. SS conceived and designed of the study. HN performed the statistical design and analysis. TM helped data collection. ST assisted in drafting the manuscript. NM performed dosimetric analysis for the manuscript. RS helped data collection. KS assisted in drafting the manuscript. RK participated the design of the study. RO assisted in drafting the manuscript. MI assisted in drafting the manuscript. KU assisted in drafting the manuscript. HS drafting of the manuscript with final approval of manuscript. All authors read and approved the final manuscript.
